# Advances in Neuromotor Stroke Rehabilitation

**DOI:** 10.1155/2014/236043

**Published:** 2014-06-19

**Authors:** Giovanni Morone, Stefano Masiero, Cordula Werner, Stefano Paolucci

**Affiliations:** ^1^Clinical Laboratory of Experimental Neurorehabilitation, Fondazione Santa Lucia, IRCCS, Via Ardeatina 306, 00179 Rome, Italy; ^2^Medical, Clinical and Experimental Sciences Doctoral School, Department of Neuroscience: Science NPSRR, University of Padua, Via Giustiniani 5, 35128 Padua, Italy; ^3^Department of Neuroscience, Unit of Rehabilitation, University of Padua, Via Giustiniani 2, 35128 Padua, Italy; ^4^Department of Neurological Rehabilitation, Medical Park Berlin, Charité-Universitätsmedizin Berlin, 13507 Berlin, Germany; ^5^Operative Unit F, Fondazione Santa Lucia, IRCCS, Via Ardeatina 306, 00179 Rome, Italy

Stroke is the second leading cause of death and the third leading cause of disability-adjusted life-years (DALYs) worldwide [1]. In the past two decades, the absolute number of people who have a stroke every year and stroke survivors and the overall global burden of stroke have been increasing [2]. This was the consequence of an increase in life expectancy and reduced mortality in the acute phase in stroke care. The public health systems and the scientific communities should seriously take into account the “pandemia” of stroke survivors [3]. From the other side, a big amount of economic resources, needed for the rehabilitation of people after stroke, is related to the increasing prevalence of patients affected by stroke sequelae. It transforms this ethical duty in an utopistic mission. There is the need of increasing rehabilitation and, at the same time, reducing its costs. New technologies for neurorehabilitation can provide modern tools for increasing efficiency. Despite the extraordinary possibilities of these new approaches, there is a lot of work for integrating them into the routinary rehabilitation programs. These devices should be considered as tools in the hands of neurorehabilitation teams usable in the framework of a rehabilitative program and not only rehabilitative per se. In fact, they should be integrated in a complex model in which the aim and the actual patients conditions concur to tailor training with multimodal conditions integrating classical and well-known conventional therapy with the new approaches, including new technological devices [4].

This complex model includes strategies for motor recruitment control, increasing performance during a task, the enhancement of patients' motivation and engagement, and trainings to empower cognitive functions, to increase cardiorespiratory fitness, to increase quality of movement, and to improve balance and motor control. Each of these specific endpoints should be addressed and concur in a different manner to the rehabilitation user-tailored program in stroke.

The efficacy of the robotic training in arm and walking neurorehabilitation after stroke has been proven in several trials; furthermore, the debate has still been open and there is no possibility at the moment to perform only machine aided training [5]. It should be noted that the machines provide the basics and the therapist's task is to implement these basics into more complex tasks; thus, no machine can replace the experienced therapists. From the other side, the high costs of such robots limit their diffusion, but the possibility that more robots can be used to treat more patients at the same time with only one physiotherapist monitoring all of them should decrease the cost of each therapy [6]. In this special issue, S. Masiero et al. analyzed the costs of the 5 years experiments on arm robotic training in hospital setting in subacute stroke, reaching this conclusion “*Robotic upper limb rehabilitation after acute stroke by NeReBot: evaluation of treatment costs.*” The increase of the rehabilitation offers in terms of number of therapies provided without increasing the costs is mandatory. In this line telerehabilitation is an important strategy when coupled to an early discharge. M. Agostini and colleagues showed us the feasibility and the efficacy of a home telerehabilitation protocol in poststroke anomia “*Telerehabilitation in poststroke anomia.*”

Video game-based therapy and the virtual reality system are playing an important role in reducing costs, especially, using commercial gaming systems, and augmenting the involvement of patients' attention and participation to the exercise/game. A paper in this special issue for the first time has shown that the balance training performed using a commercial console is more effective than the conventional therapy: “*The efficacy of balance training with video game-based therapy in subacute stroke patients: a randomized controlled trial.*” Furthermore, the development of a patients' tailored system with specific exercises and specific augmented feedback as described in the paper of P. Kiper et al. “*Reinforced feedback in virtual environment for rehabilitation of upper extremity dysfunction after stroke: preliminary data from a randomized controlled trial*” provides a next step toward this aim.

Quantitative assessment is also important for providing a tailored rehabilitation. The study of M. Manca et al. identified five clusters characterizing the gait dysfunction of patients with stroke “*Gait patterns in hemiplegic patients with equinus foot deformity.*” Similarly, I. Aprile et al. have identified an upper limb kinematic analysis for assessing alterations in reaching function, providing more chances to train each patient in a specific tailored way “*Kinematic analysis of the upper limb motor strategies in stroke patients as a tool towards advanced neurorehabilitation strategies: a preliminary study.*”

Among the novel rehabilitation approaches, the action observation is another one of interest. The observation of actions performed by other people activates in the perceiver the same neural structures responsible for the actual execution of the same actions exploiting the neurophysiological mechanism for the recovery of motor impairment as shown in the paper of P. Sale and colleagues “*Action observation therapy in the subacute phase promotes dexterity recovery in right-hemisphere stroke patients.*”

The complex model for treating patients with stroke, in the last years, involved also cardiovascular fitness, in chronic [7], as well as in subacute, phase [8]. The energy cost of walking assessed by a breath to breath method could be expensive involving machines with high costs and not easy to use; conversely, the physiological cost index is easy to use and of low cost. A. S. DeLussu and colleagues have proved the validity of the physiological cost index for assessing the energy cost during walking training on floor and on robotic gait training in subacute stroke “*Concurrent validity of physiological cost index in walking over ground and during robotic training in subacute stroke patients.*”

Finally, the attention to the sleepiness problems and the correlation with the disabilities is increasing after stroke, but little is known about the sleep and daytime sleepiness of chronic stroke patients as an important factor conditioning patients' ability in activity of daily living. The paper of K. Herron et al. explored the sleep and sleepiness in a chronic stroke population with sustained physical deficits “*Quantitative electroencephalography and behavioural correlates of daytime sleepiness in chronic stroke.*”

All the studies reported in this special issue showed the complexity of the neurorehabilitation of people who suffered from stroke and the consequent need of a complex tailored rehabilitation that is not only an economic burden but also an ethical issue. The way a nation cares for people with disabilities is an indicator of its progress.

*Giovanni Morone* *Stefano Masiero* *Cordula Werner* *Stefano Paolucci*
